# Supplementary material for: Adaptive landscape flattening allows the design of both enzyme: Substrate binding and catalytic power
Source: PLoS Comput Biol. 2020 Jan 9;16(1):e1007600. doi: 10.1371/journal.pcbi.1007600 (PMC7041857; doi:10.1371/journal.pcbi.1007600)
Supplement: S1 File — This file includes a short theoretical derivation, some explanation of force field parameters, atomic charges for the MetRS transition state ligands, a figure showing the MetRS:AnL complex structure, and MetRS:MetAMP binding free energy results obtained with the FDBLK solvent model. (PDF) [file pcbi.1007600.s001.pdf]

Supplementary Appendix:
Adaptive landscape flattening allows the design of both enzyme:substrate binding and catalytic power

Vaitea Opuu, Giuliano Nigro, Thomas Gaillard, Emmanuelle Schmitt, Yves Mechulam & Thomas Simonson*
Laboratoire de Biochimie, Ecole Polytechnique, Palaiseau, France

Relation between free energies with and without the bias potential

The free energy of a sequence S is denoted $G_X(S)$, where X indicates the apo or holo system. $G_X(S)$ is defined by a Boltzmann average over all possible conformations r:

$$e^{-\beta G_X(S)} = \int e^{-\beta E_X(S,r)}\, dr \tag{1}$$

where β is the inverse of the thermal energy kT. The bias potential $E^B(S)$ depends only on the sequence, not r. Therefore, for the free energy $\tilde{G}_X(S)$ in the presence of the bias, we have

$$e^{-\beta \tilde{G}_X(S)} = \int e^{-\beta(E_X(S,r)+E^B(S))}\, dr = e^{-\beta E^B(S)} \int e^{-\beta E_X(S,r)}\, dr = e^{-\beta E^B(S)} e^{-\beta G_X(S)} \tag{2}$$

which gives Eq. (7) in the main text.

Force field parameters for AnL and MetAMP

Force field information is given in the files AnL.ff and MetAMP.ff. Each file contains the "topology" or 2D structure of each molecule, including the atomic charges. This is followed by the energy parameters for covalent bonds, angles, dihedrals and impropers, van der Waals and Generalized Born terms. The data are in the format of the Proteus software, with comments for clarity. With minor reformatting, they can also be read by XPLOR, CHARMM and NAMD.

Atomic charges for the Met + ATP → MetAMP + PP$_i$ transition state

The transition state charges are given below, in the form of a Proteus topology file:

```
ATOM MG   TYPE=MG  CHARGE= 1.5000

ATOM N    TYPE=N3  CHARGE=-0.3025
ATOM HN1  TYPE=H   CHARGE= 0.2770
ATOM HN2  TYPE=H   CHARGE= 0.2770

ATOM O2A  TYPE=O2  CHARGE=-0.7016
ATOM O3A  TYPE=OA  CHARGE=-0.8680
ATOM O5'  TYPE=OS  CHARGE=-0.4478
ATOM C5'  TYPE=CT  CHARGE= 0.0558
ATOM H5'1 TYPE=H1  CHARGE= 0.0679
```

```
ATOM HN3  TYPE=H   CHARGE= 0.2770
ATOM CA   TYPE=CT  CHARGE= 0.0204
ATOM HA   TYPE=HP  CHARGE= 0.0741
ATOM CB   TYPE=CT  CHARGE= 0.0297
ATOM HB2  TYPE=HC  CHARGE= 0.0195
ATOM HB3  TYPE=HC  CHARGE= 0.0195
ATOM CG   TYPE=CT  CHARGE=-0.0027
ATOM HG2  TYPE=H1  CHARGE= 0.0394
ATOM HG3  TYPE=H1  CHARGE= 0.0394
ATOM SD   TYPE=S   CHARGE=-0.2782
ATOM CE   TYPE=CT  CHARGE=-0.0580
ATOM HE1  TYPE=H1  CHARGE= 0.0638
ATOM HE2  TYPE=H1  CHARGE= 0.0638
ATOM HE3  TYPE=H1  CHARGE= 0.0638
ATOM C    TYPE=C   CHARGE= 0.9610
ATOM O    TYPE=O   CHARGE=-0.7856
ATOM OXP  TYPE=OA  CHARGE=-0.7517

ATOM PG   TYPE=P   CHARGE= 1.4463
ATOM O1G  TYPE=O3  CHARGE=-1.0141
ATOM O2G  TYPE=O3  CHARGE=-0.9438
ATOM O3G  TYPE=O3  CHARGE=-0.9153
ATOM PB   TYPE=P   CHARGE= 1.5390
ATOM O1B  TYPE=O2  CHARGE=-0.9582
ATOM O2B  TYPE=O2  CHARGE=-0.8900
ATOM O3B  TYPE=OS  CHARGE=-0.6252
ATOM PA   TYPE=P5  CHARGE= 1.2530
ATOM O1A  TYPE=O2  CHARGE=-0.6138
ATOM H5’2 TYPE=H1  CHARGE= 0.0679
ATOM C4’  TYPE=CT  CHARGE= 0.1065
ATOM H4’  TYPE=H1  CHARGE= 0.1174
ATOM O4’  TYPE=OS  CHARGE=-0.3548
ATOM C1’  TYPE=CT  CHARGE= 0.0394
ATOM H1’  TYPE=H2  CHARGE= 0.2007
ATOM N9   TYPE=N*  CHARGE=-0.0251
ATOM C8   TYPE=CK  CHARGE= 0.2006
ATOM H8   TYPE=H5  CHARGE= 0.1553
ATOM N7   TYPE=NB  CHARGE=-0.6073
ATOM C5   TYPE=CB  CHARGE= 0.0515
ATOM C6   TYPE=CA  CHARGE= 0.7009
ATOM N6   TYPE=N2  CHARGE=-0.9019
ATOM HN61 TYPE=H   CHARGE= 0.4115
ATOM HN62 TYPE=H   CHARGE= 0.4115
ATOM N1   TYPE=NC  CHARGE=-0.7615
ATOM C2   TYPE=CQ  CHARGE= 0.5875
ATOM H2   TYPE=H5  CHARGE= 0.0473
ATOM N3   TYPE=NC  CHARGE=-0.6997
ATOM C4   TYPE=CB  CHARGE= 0.3053
ATOM C3’  TYPE=CT  CHARGE= 0.2022
ATOM H3’  TYPE=H1  CHARGE= 0.0615
ATOM C2’  TYPE=CT  CHARGE= 0.0670
ATOM H2’  TYPE=H1  CHARGE= 0.0972
ATOM O2’  TYPE=OH  CHARGE=-0.6139
ATOM HO2’ TYPE=HO  CHARGE= 0.4186
ATOM O3’  TYPE=OH  CHARGE=-0.6541
ATOM HO3’ TYPE=HO  CHARGE= 0.4376
```

Structure of the SLL:AnL complex from Proteus and experiment

Figure A: Complex between AnL and the SLL MetRS mutant. Red: lowest-energy Proteus structure; green: X-ray. Side chains close to the ligand; the 4 largest deviations are labeled.

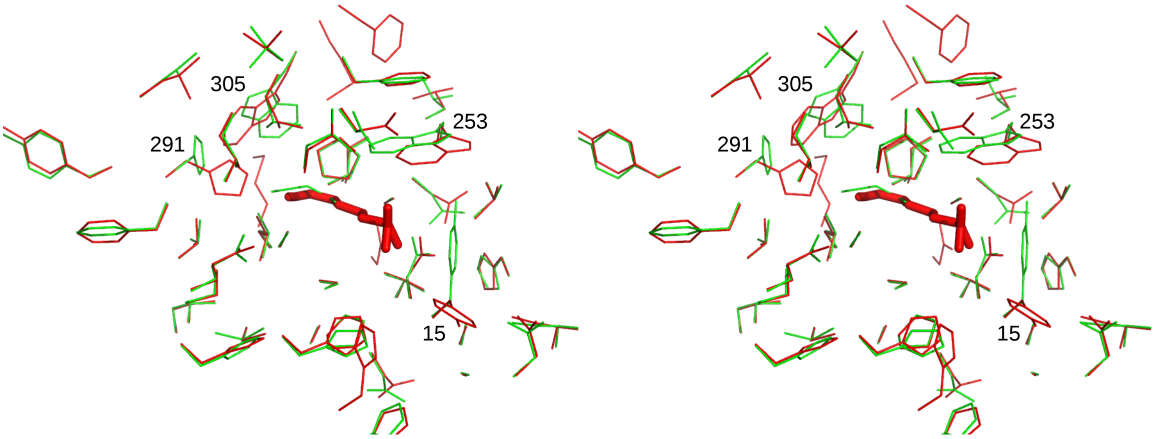

MetRS:MetAMP binding free energies with the FDBLK solvent model

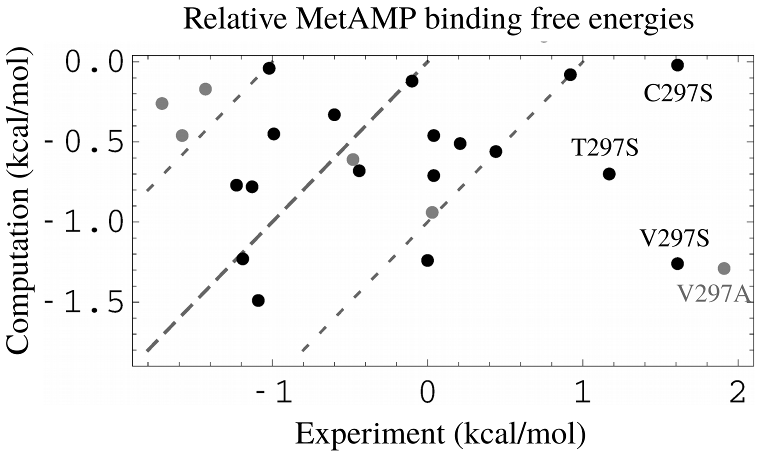

Figure B: MetRS:MetAMP binding free energies relative to WT, with the FDBLK solvent. Data are for 27 point mutations, as in the main text (Fig. 4). The largest errors are labeled.
